# Struvite: a slow-release fertiliser for sustainable phosphorus management?

**DOI:** 10.1007/s11104-015-2747-3

**Published:** 2015-12-11

**Authors:** Peter J. Talboys, James Heppell, Tiina Roose, John R. Healey, Davey L. Jones, Paul J. A Withers

**Affiliations:** School of Environment, Natural Resources and Geography, Deiniol Road, Bangor, Gwynedd LL57 2UW UK; Institute for Complex Systems Simulation, University of Southampton, Southampton, SO17 1BJ UK; Faculty of Engineering and the Environment, University of Southampton, Southampton, SO17 1BJ UK

**Keywords:** Phosphorus, Struvite, Wheat, Buckwheat, Fertiliser, Root system modelling, Organic acids

## Abstract

**Background and aim:**

Recycled sources of phosphorus (P), such as struvite extracted from wastewater, have potential to substitute for more soluble manufactured fertilisers and help reduce the long-term threat to food security from dwindling finite reserves of phosphate rock (PR). This study aimed to determine whether struvite could be a component of a sustainable P fertiliser management strategy for arable crops.

**Methods:**

A combination of laboratory experiments, pot trials and mathematical modelling of the root system examined the P release properties of commercial fertiliser-grade struvite and patterns of P uptake from a low-P sandy soil by two different crop types, in comparison to more soluble inorganic P fertilisers (di-ammonium phosphate (DAP) and triple super phosphate (TSP)).

**Results:**

Struvite had greatly enhanced solubility in the presence of organic acid anions; buckwheat, which exudes a high level of organic acids, was more effective at mobilising struvite P than the low level exuder, spring wheat. Struvite granules placed with the seed did not provide the same rate of P supply as placed DAP granules for early growth of spring wheat, but gave equivalent rates of P uptake, yield and apparent fertiliser recovery at harvest, even though only 26 % of struvite granules completely dissolved. Fertiliser mixes containing struvite and DAP applied to spring wheat have potential to provide both optimal early and late season P uptake and improve overall P use efficiency.

**Conclusions:**

We conclude that the potential resource savings and potential efficiency benefits of utilising a recycled slow release fertiliser like struvite offers a more sustainable alternative to only using conventional, high solubility, PR-based fertilisers.

**Electronic supplementary material:**

The online version of this article (doi:10.1007/s11104-015-2747-3) contains supplementary material, which is available to authorized users.

## Introduction

Phosphorus (P) is a plant nutrient essential for cellular function and plant growth. In agriculture, P is often applied in the form of processed phosphate salt granules that dissolve into soil pore water and increase plant P uptake. Alongside other nutrient inputs, application of these P fertiliser salts has dramatically raised agricultural productivity in developed countries during the 20th century, but has also contributed to widespread eutrophication (Dodds et al. 2009; Withers et al. 2014a). Conventional mineral P fertilisers are manufactured from finite reserves of phosphate rock (PR). Global reserves of good quality PR are dwindling, becoming more expensive and are now concentrated in only a few countries (Reijnders 2014). This raises concerns over the affordability of P fertilisers and threatens future food security, especially in those countries that have little or no PR reserves (Cordell and Neset 2014; Elser et al. 2014). The high dependency of agriculture on PR, and the growing issue of eutrophication of our inland and coastal waters from P leakage along food production chains, has prompted a renewed and urgent interest in the concept of closing the P cycle by recovering and recycling P (Elser and Bennett 2011; Withers et al. 2015b). For example, the P extracted from sources such as animal manures (Greaves et al. 1999), source-separated urine (Wilsenach et al. 2007), or human wastewater (Le Corre et al. 2009) can be processed into user-friendly fertiliser products for agriculture, subject to technological and economic constraints (Lederer et al. 2014; Withers et al. 2015a). Fertiliser-grade struvite (NH_4_MgPO_4_·6H_2_O) is one recovered P product that is easy to spread, has a high P content and can be produced with minimal heavy metal contamination (e.g. Antonini et al. 2012). Struvite is frequently produced as a by-product of wastewater treatment; at locations within treatment plants where there are rapid pressure changes, it forms a scale on lines and clogs pipes (Jaffer et al. 2002). However, controlled struvite precipitation can be triggered in specialised reactors by manipulation of the sludge digestion process to overcome these problems (Baur 2009). This can produce struvite granules that are useable as a magnesium ammonium phosphate fertiliser product for agriculture, whilst also reducing plant effluent P concentrations discharged to watercourses (Baur 2009; Schauer et al. 2011).

In addition to savings in resource use, struvite provides potential efficiency savings and environmental benefits over conventional fertilisers due to its low solubility (Bhuiyan et al. 2007; Cabeza et al. 2011; Massey et al. 2009). Conventional mineral P fertilisers are readily soluble and can cause high P concentrations in land runoff when rain falls soon after fertiliser application (Hart et al. 2004), with increased risk of eutrophication of receiving waterbodies. When incorporated into the soil, highly water-soluble P fertilisers also lead to high soil solution P concentrations for early crop growth, but this solution P quickly becomes adsorbed and immobilised onto soil particle surfaces (Chang and Chu 1961; Holford and Mattingly 1975; Barrow and Debnath 2014). This results in a much more limited P supply to crops in the later stages of growth when crop P demand is high (Veneklaas et al. 2012). Struvite, as a less soluble “slow release” fertiliser, could provide a longer term source of P for crop growth than readily soluble forms of P, thus more closely matching the plant’s demand for P later in the growing season and increasing its efficiency of use (Withers et al. 2014b). The slower dissolution of struvite could also reduce the amount of fertiliser P that becomes adsorbed on to soil particles, or released to land runoff. These benefits could therefore potentially be used to either increase crop yields, or allow reduced application rates of P whilst maintaining or increasing yields with minimum environmental impact: all of which would be economically advantageous to the agricultural industry as it moves towards sustainable intensification in the future.

Although struvite is only slightly soluble in water (1–5 %, Achat et al. 2014b; Cabeza et al. 2011), much previous laboratory-based work has suggested that struvite is as effective as highly soluble mineral P fertiliser as a source of P for plants (Achat et al. 2014a, b; Antonini et al. 2012; Bonvin et al. 2015; Cabeza et al. 2011; Johnston and Richards 2003; Massey et al. 2009). However, the reasons for this apparent dichotomy and the particular mechanisms of struvite P dissolution still remain unclear. Its low water-solubility might suggest that the release of P may be too slow for early crop growth, although Bonvin et al. (2015) recently found that P uptake rates from synthetic urine-derived struvite were fairly constant over a 30–72 day growing period. Provision of P uptake during early growth stages is critical for plant growth and establishment and for optimizing crop yields (Boatwright and Viets 1966; Grant et al. 2001; Nadeem et al. 2011). To overcome soil P immobilisation, inorganic P fertilisers are often placed close to the germinating seed to encourage root growth and optimise plant P uptake. Numerous experiments have shown a unique yield advantage from the placement or banding of P fertiliser in soils with limited P availability due to enhancement of solution P content in the immediate rooting zone (Flaval et al. 2014; Jing et al. 2010; Randall and Hoeft 1988; Wager et al. 1986). This agronomic practice is increasingly viewed as an important component of more sustainable production systems on low-P soils (Rowe et al. 2015; Withers et al. 2014b). However, the effectiveness of placed struvite has not been evaluated in previous pot studies.

In this study, we investigated in laboratory and pot experiments whether commercial fertiliser-grade struvite represents a more efficient and sustainable alternative fertiliser to conventional PR-based fertilisers by answering the following questions. (1) Given that struvite is more soluble in low pH conditions (Ohlinger et al. 1998; Massey et al. 2009), does pH in the range found in UK arable soils (5.5–8.0) significantly affect its potential as a P fertiliser? (2) Do compounds exuded from plant roots, such as organic acids, affect the dissolution of struvite P, and does this influence its plant uptake? (3) Is replacing the application of readily soluble P fertiliser with struvite beneficial for plant P uptake? (4) Does the slow release of P from struvite negatively impact early plant growth, and can this be compensated for by adding fertiliser mixtures of soluble P and struvite? We uniquely included mathematical modelling to help identify optimum mixtures of soluble P and struvite for optimum P uptake because of the ability of such models to simulate root uptake of nutrients (Ge et al. 2000; Grant and Robertson 1997; Lynch and Brown 2001). The plant and soil models of Heppell et al. (2015) and Roose and Fowler (2004) allow field scale predictions by estimating plant P uptake per soil surface area, thereby converting the results from the pot experiments into approximate field-scale predictions of struvite effectiveness. The use of modelling alongside pot experiments in this manner enabled the results of short duration pot experiments to be projected to a full growing season’s results.

## Materials and methods

### Struvite source

Struvite granules commercially distributed under the trade name Crystal Green® were provided by Ostara Nutrient Recovery Technologies Inc. These white granules have been classified as fertiliser grade material in the UK and measured approximately 2.4 mm in diameter. Crystal Green is precipitated from wastewater using the WASSTRIP™ (Baur 2009) and PEARL® processes and the granules contain >99 % struvite (NH_4_MgPO_4_^.^6H_2_O) equivalent to 12 % P (28 % P_2_O_5_).

### Struvite solubility assays

Reactions to test struvite solubility under varying pH conditions, using different counter-ions and in the presence of different organic acids including nil addition controls, were performed in 1.5 ml tubes filled with 1 ml deionised water. The deionised water was buffered with 0.01 M Di-sodium EDTA and 0.01 M NaCl to stabilise both the pH and salinity during the early period of dissolution. There were three experimental replicates per treatment. Solution pH was adjusted to between 5.5 and 8.0 using HCl and NaOH. Struvite granules weighing between 30 and 40 mg (3.6–4.8 mg P) were then submerged in 1 ml of these solutions per replicate, and the resulting reaction mixtures were kept at room temperature. Solution aliquots of 5 μl were taken at successive time intervals, and their P content was determined using the ascorbate/molybdate blue method of Murphy and Riley (1962). Changes in solution P concentrations with time were plotted using a modified Mitscherlich equation, which has been previously used to model PR dissolution (Mackay et al. 1986):

1 $$ c=\mathrm{a}\left(1-{b}^t\right) $$

Where *b* is the curvature coefficient, *a* is the asymptote (equilibrium P concentration), *c* is the solution P concentration in mM and *t* is time elapsed in days.

Initial dissolution rates were calculated from the differential of Eq. 1:

2 $$ \frac{dc}{dt}=-aln(b){e}^{tlnb} $$

For the assays including organic acids or counter ions, all replicates were adjusted to pH 6.0 after the addition of either acetic acid, malic acid, oxalic acid, citric acid, MgCl_2_, NH_4_Cl or K_2_HPO_4_. Using the same method as for the pH assays, 30–40 mg of struvite granules were submerged in 1 ml of solution and 5 μl aliquots taken at successive time intervals. Organic acids were added at concentrations of 1 mM and each counter ion (i.e. Mg^2+^, NH_4_^+^ and PO_4_^3−^) was added at concentrations of 1 μM, 10 μM, 100 μM and 1 mM. The organic acids selected are known to be exuded by plant root systems (Jones 1998), and are mono- (acetic acid), di- (malic acid, oxalic acid) or tri-valent (citric acid). The counter ions are those present in struvite and therefore might be expected to affect dissolution rates.

To test whether struvite would dissolve faster in the presence of an infinite ion sink, an individual pre-weighed struvite granule was incubated with 10 g of a mixed cation-anion exchange resin in a PST-1 resin capsule (H^+^/OH^−^ saturated; Unibest International Corporation, Pasco, WA, USA) in 50 ml of distilled water. A control treatment with a struvite granule in water but with no resin was also included, with five replicates of each treatment. Struvite granules were recovered after 10 days, dried and re-weighed. Differences in granule dissolution were recorded as the percentage weight loss over the 10-day period.

### Pot experiments

Three pot experiments tested the effect of different P fertilisers (di-ammonium phosphate (DAP), triple super phosphate (TSP) or struvite) on plant growth and P uptake. The same soil, variety and experimental conditions were used for all three experiments and all the fertilisers were applied as commercial–grade granules placed beneath the soil surface.

1. **30-day pot experiment:** to confirm laboratory assay results suggesting that organic acids might enhance struvite P solubility, *Triticum aestivum* (spring wheat, a low organic acid exuder) and *Fagopyrum esculentum* (buckwheat, a high organic acid exuder) were both grown in 8 cm-diameter 6.5 cm-deep pots filled with 300 g of a sandy loam Eutric Cambisol soil receiving either nil P (untreated control) or 35 kg P ha^−1^ as either struvite or DAP. The soil (from Henfaes Research Farm, Bangor University, Abergwyngregyn, UK) had a low Olsen P concentration of 13 mg kg^−1^ which provided a ‘P-limiting’ environment for plant growth according to current recommendation systems used in England and Wales (Defra 2010). The soil had a P sorption capacity of 150 mg kg^−1^, and a pH of 6.0. The P fertiliser granules were placed in one location 1 cm along the radius from the seed (in the centre of the pot), and 2.5 cm below the soil surface. The amount of fertiliser P added is the recommended amount of P required for a high-yielding wheat crop on a low-P soil (Defra 2010), and equated to 17.6 mg P per pot. Five replicates were planted for each treatment/crop combination, but poor germination meant that *n* = 4 for each, apart from spring wheat grown with struvite where *n* = 5.
  Three seeds of each crop were sown initially in the same location but, at crop emergence, the excess seedlings were removed to leave only the largest seedling in each pot. The pots were kept in a heated greenhouse with artificial lighting set to produce an air temperature of 20 °C and a minimum 16 h of day length. Soil moisture was measured gravimetrically, and maintained at 80 % of soil water holding capacity. To ensure that P was the only limiting macronutrient, pots received 30 mg N pot^−1^ (equivalent to 60 kg ha^−1^ N as 1 M NH_4_NO_3_ solution) and 30 mg K pot^−1^ (equivalent to 60 kg ha^−1^ K_2_O as 1 M KCl solution). Micronutrients were supplied in a weekly application of 10 ml of a solution containing: 5 mM CaCl_2_; 2 mM MgSO_4_; 765 nM ZnSO_4_; 320 nM CuSO_4_; 46.3 μM H_3_BO_3_; 497 μM Na_2_MoO_4_; 9.14 μM MnCl_2_ and 38.7 μM Fe.EDTA (all Sigma Aldrich, Poole, UK).
  At harvest, the whole plant was extracted from the pot and the root system washed in deionised water to remove any soil particles. All plant tissue was then dried at 85 °C overnight, weighed, and dry-ashed (550 °C, 16 h). The residue was dissolved in 0.5 M HCl and the P content was determined (Murphy and Riley 1962). Any remaining struvite granules were extracted from the soil, air-dried, any adhering soil particles brushed off, and re-weighed at the end of each experiment. There were no discernible DAP granules remaining at the end of the experimental period. Apparent fertiliser P recovery was calculated as the plant P content in the fertilized treatments minus plant P content in the control treatment divided by the applied fertiliser P.

2. **90-day pot experiment:** to assess the longer-term effectiveness of struvite granules as a fertiliser source relative to soluble P fertiliser granules over a full crop growing season, spring wheat was grown in 11 cm-diameter 30 cm-deep drainpipes filled to the top with 3 kg of soil receiving either struvite (*n* = 5), TSP (*n* = 5) or nil P (untreated controls, *n* = 8). TSP was used instead of DAP simply because this was the only source of soluble P fertiliser available at the time of the experiment. Fertiliser P was added in the same areal amount (i.e. 35 kg P ha^–1^, equating to 33.3 mg P per pot) and placed in exactly the same way as for the 30-day pot experiment, except that the granules were placed at 5 cm depth below the soil surface to account for the pot’s increased depth. Fertiliser N and K fertilisation regimes were applied as per current recommendations (Defra 2010): pots received 57 mg pot^−1^ N (equivalent of 60 kg ha^−1^ N as 1 M NH_4_NO_3_ solution) and 57 mg K pot^−1^ (equivalent 60 kg ha^−1^ K_2_O as 1 M KCl solution) at seedling emergence, and another 57 mg pot^−1^ N (60 kg N ha^−1^) applied at the stem extension stage. Trace elements were applied in the same manner as for the 30-day experiment. At harvest, the whole crop was harvested, roots washed, seed removed, components separately weighed, dried and analysed for P content. Any remaining intact struvite granules were similarly recovered, cleaned and weighed as for the 30-day experiment.
3. **36-day pot experiment:** to overcome potential shortfalls in P supply from struvite during early crop growth, spring wheat was grown in 8 cm-diameter 6.5 cm-deep pots filled with 300 g of soil in exactly the same manner as for the 30-day pot experiment, but with varying ratios of applied struvite:DAP of 100:0 (*n* = 3), 30:70 (*n* = 3), 20:80 (*n* = 3), 10:90 (*n* = 4), 0:100 (*n* = 3) together with untreated controls (*n* = 4). The amount of P added to the fertiliser treatments was kept constant at 17.6 mg P per pot. Four replicates were planted, but variable germination rates meant *n* = 3 in some cases. Dry matter yield and P offtake were measured and struvite granules recovered in the same way as for the 30-day pot experiment.

### Modelling P uptake from a growing root system

A root system model that has previously been used to simulate P uptake from wheat root systems (i.e. scaling P uptake per soil surface area to a desired pot or field scale) by accounting for P-fertiliser inputs and the resulting alterations in root branching (Heppell et al. 2015; Roose and Fowler 2004) was calibrated for the same soil and struvite:DAP fertiliser mixtures used in the 36-day pot experiment. To account for the variations in solubility of the different P fertilisers, a source term was added at a given soil depth to the nutrient conservation equation used in the model. By varying the rate of fertiliser source over time, the model attempted to mimic the effect of different combinations of soluble P (in this case DAP) and slow release P (in this case struvite) fertiliser on P uptake. The model was set up so that fertiliser P was released at depths between 0 and 10 cm below the seed, with peak release rates at 5 cm and a linear decline to zero at 0 and 10 cm. In order to match the release of fertiliser P in the model to the dissolution of DAP and struvite, a set of dissolution curves were produced. This was done in exactly the same way as for the previous solubility assays by placing a granule of DAP or struvite into 1 ml of deionised water, and measuring the solution P concentration over time (Murphy and Riley 1962). This was performed in triplicate for both fertilisers, with granule weights ranging from 30 to 40 mg as used in the laboratory assays and pot trials. Dissolution rates from in vitro in absence of soil is adequate for the model as the model already takes into account the buffering effect of soil P binding phases on P concentrations in the soil solution via a buffer power parameter determined for the soil (Heppell et al. 2015).

**Fig. 1:**
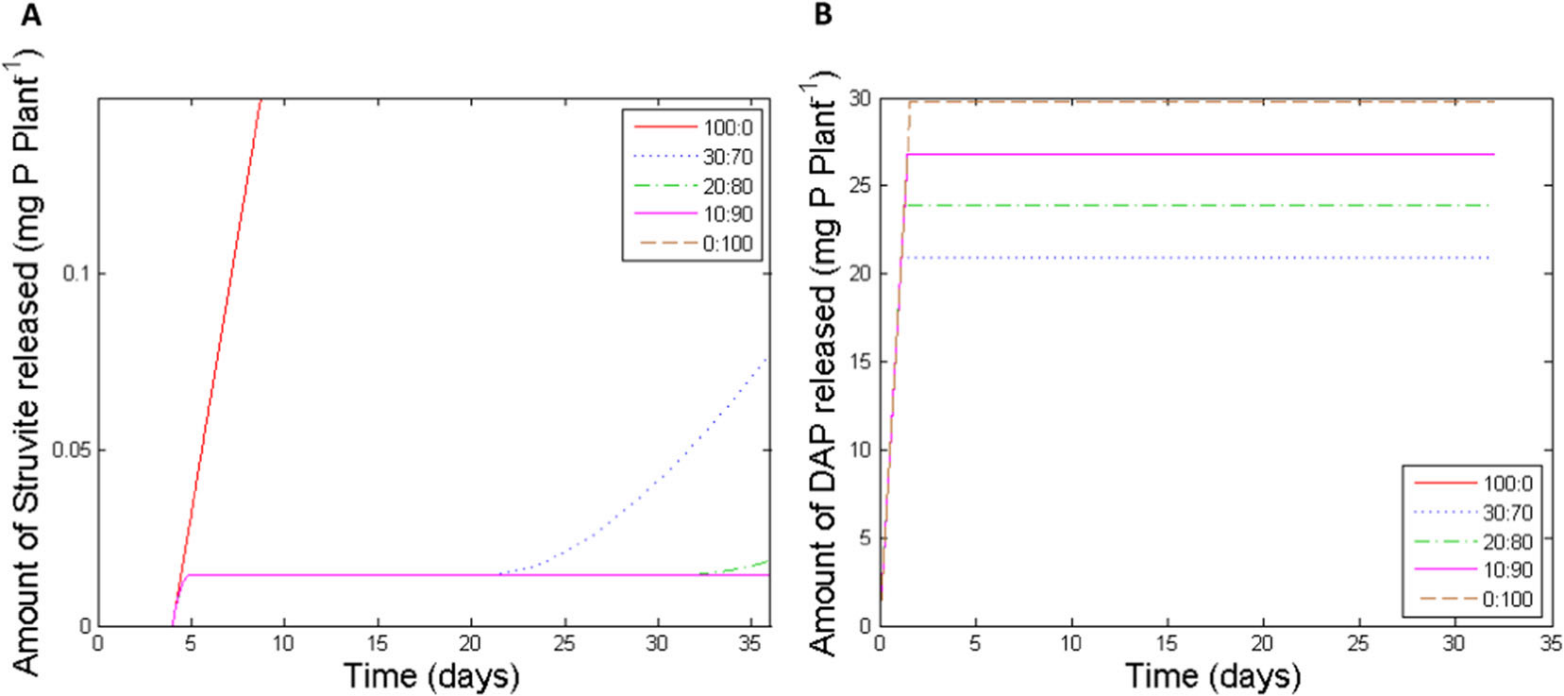
Model simulations of P concentrations in the soil solution arising from: **a** struvite dissolution, and **b** diammonium phosphate (DAP) dissolution after placing mixtures of struvite:DAP at 5 cm below the soil surface. The simulations provided the baseline for model predictions of P uptake in the 36-day and 90-day pot experiments

The DAP and struvite P dissolution rates were first fitted by a modified Mitscherlich equation (Eq. 1). The total release of P (μmol) was fitted against time, and integrated to acquire the release rate of P over time (μmol *day*^*−1*^). Plots of concentration of P (*mol l*^−1^) against release rate of P (μmol *day*^*−1*^) were best fitted by a straight line. For DAP the equation was y = (x-0.4253)/(−0.003712), whereas for struvite the equation was y = (x-0.001225)/(−0.004200). Therefore, at a given soil solution P concentration, P was released from each fertiliser at a realistic rate. When the soil solution P concentration rose above the point where P dissolution reached equilibrium for a fertiliser granule, its P release was halted. For DAP and struvite these values were calculated to be 13.6 and 0.04 mg l^−1^ respectively. The two orders of magnitude difference in these values reflects the large difference in solubility between the two fertiliser types. The resulting P release rates for mixtures of DAP and struvite fertilisers as tested in the 36-day pot experiment can be seen in Fig. 1, which illustrates struvite release rates halting due to DAP release (increasing local P concentration). Without DAP in the mixture, struvite continues to release P throughout the simulation and the local P concentration does not reach the struvite P release rate limit.

To establish an initial soil P concentration within the model (denoting the combined sorbed and solution P in the system), air-dried samples were extracted according to the Olsen P method (Olsen et al. 1954), and P was determined by colour (Murphy and Riley 1962). The extracted value for soil-available P was 13 mg P kg^−1^ for Olsen P at the start of the study. To account for previously observed increases in root proliferation of this wheat variety following fertiliser application (Talboys et al. 2014), the maximum root branching rates for plants under DAP, struvite and control (no fertiliser) treatments were calculated from images of intact 36-day-old *T. aestivum* root systems grown as described for the pot experiments with placed P treatments (Supplementary Figure 1). We matched plant root chemotropism (as seen in the experimental data) by using a constant root branching rate for the untreated control simulations, as has been done previously (Heppell et al. 2015; Roose and Fowler 2004), and a branching rate that decreased exponentially with increasing depth from a maximum for fertilised treatments. In all simulations, the volume of the root system at 90 days was kept constant so that the final simulation results were comparable in terms of efficacy of treatments. The 36-day pot experiment provided plant P uptake values for calibrating the model. In the model we set an initial root length between 5 and 10 cm, which took approximately four days to reach in the experiments. The model was therefore run for 32 days to mirror the pot experimental data for this time-point. The model simulations used identical DAP and struvite treatments to the 36-day pot experiment described above, and were then run to a 90 day time-point to assess the effects of these treatments on total P uptake at harvest.

## Results

### Struvite P release

**Fig. 2:**
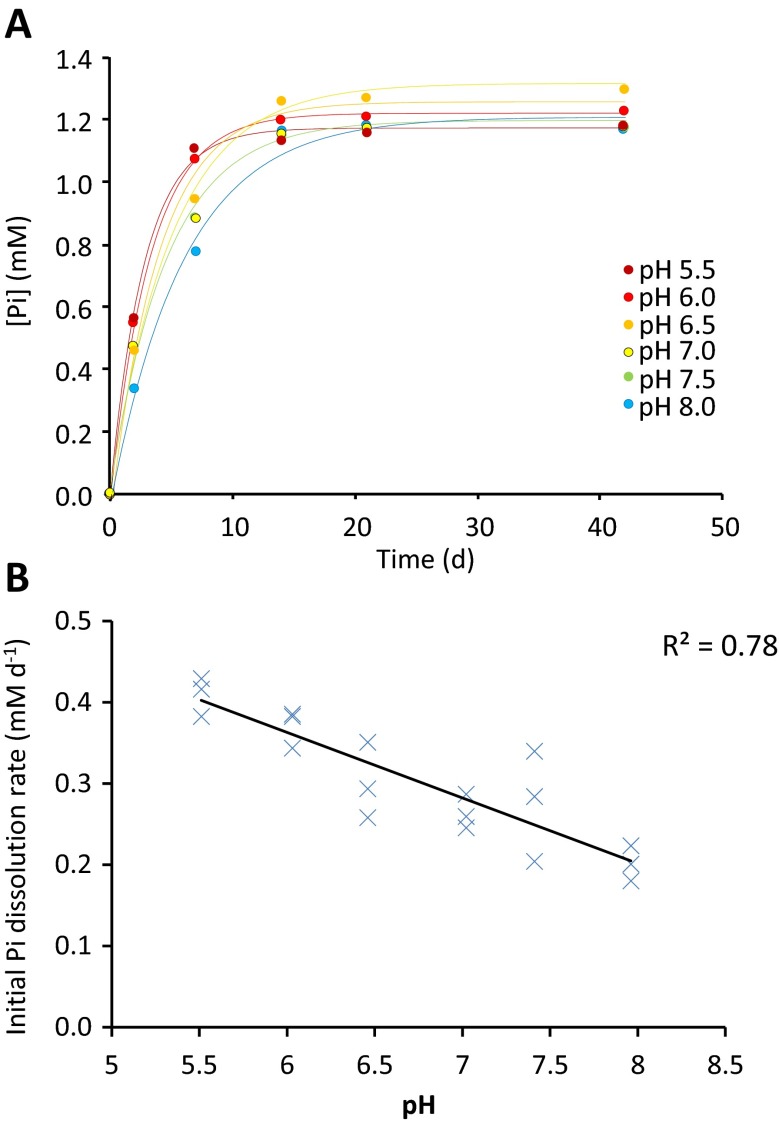
The effects of solution pH on 2.4 mm struvite granule dissolution. **a** Changes in solution P concentration over time, with the curve f(x) = a(1-b^x^) fitted for each initial pH. All three replicates were used to fit each curve. **b** Initial dissolution rates calculated from the curve fitting depicted in A decreased significantly with increasing pH

The solubility curves for struvite P over the pH range 5.5–8.0 provided good fits (r^2^ > 0.9 for each replicate) to the modified Mitscherlich equation (Fig. 2a). The initial struvite P dissolution rate showed a strong negative correlation with increasing initial pH (r^2^ 0.78, Fig. 2b), but there was no discernible impact of initial pH on the equilibrium P concentration in solution at the end point of the experiment at 42 days. Upon commencement of the assay, the pH of the replicates starting at 5.5, 6.0 and 6.5 increased to pH 6.9–7.1 after 2 days, those with initial pH 7.0 increased to pH 7.2–7.4, those with initial pH 7.5 increased to 7.5–7.6, and those with initial pH 8.0 remained unchanged. As struvite dissolved very slowly, solution pH therefore increased.

When the initial concentration of the PO_4_^3−^ counter-ion was varied at constant initial pH, there was a significant inhibitory effect of increasing initial PO_4_^3−^ concentration on both initial dissolution rate and equilibrium P concentration (*p* < 0.05) (Supplementary Fig. 2C, F). Increased NH_4_^+^ concentration also significantly reduced the end-point equilibrium P concentration (*p* < 0.05) (Supplementary Fig. 2D). Initial P dissolution rate was not significantly affected by altering the starting NH_4_^+^ or Mg^2+^ concentration, with Mg^2+^ also having no significant effect on the equilibrium P concentration (*p* > 0.05) (Supplementary Fig. 2A, B, E). In all cases, the equilibrium P concentration was reached with less than 1 % of the P being released from the struvite granule. In a separate experiment, the inclusion of an infinite resin sink for Mg^2+^, NH_4_^+^ and PO_4_^3−^ did not significantly increase P dissolution from the struvite granules: percentage weight loss was 9.6 % (s.e.m. 2.12) with the resin sink and 6.9 % (s.e.m. 0.46) without the resin.

**Fig. 3:**
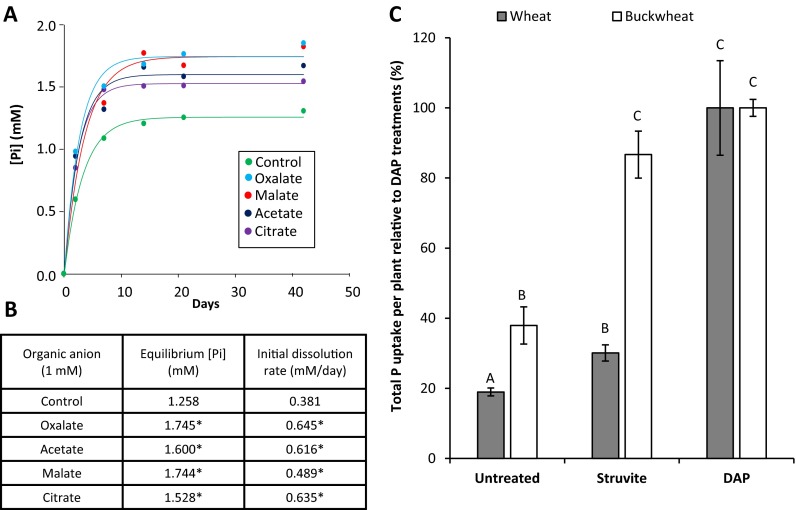
Organic acid promotion of struvite P dissolution and uptake. **a** The effect of 1 μM oxalic acid, malic acid, acetic acid, or citric acid on struvite P dissolution fitted to the curve f(x) = a(1-b^x^). **b** The initial P dissolution rate and final equilibrium P concentrations were calculated by fitting the curve f(x) = a(1-b^x^) to each replicate. Asterisks represent values that are significantly different from the controls using student’s t-test (*p* < 0.05). **c** A 30-day pot experiment growing seedlings of *Triticum aestivum* and *Fagopyrum esculentum* in low-P sandy soil; P was applied at 17.6 mg P pot^−1^ (35 kg P ha^−1^) in the form of diammonium phosphate (DAP) or struvite alongside untreated controls. Values are the total P uptake per plant, divided by the average P uptake per plant of the DAP treatments for that species. This is to make the values fit better on one axis, as the buckwheat P uptake is universally higher. Total P uptake relating to 100 % were 0.711 mg for *T. aestivum* and 2.267 mg for *F. esculentum.* Letters represent values that are significantly different from the DAP positive controls for each species using student’s t-test (*p* < 0.05). Error bars are standard errors of the mean

The addition of four organic acids commonly exuded by plant roots (Jones 1998), with equal pH in comparison to controls with no organic acid, resulted in marked increases in struvite P solubilisation (Fig. 3). Both the initial rate of dissolution and the equilibrium P concentration (Fig. 3b) showed significant increases (by up to 69 % and 39 %, respectively) in the presence of 1 mM acetate, oxalate, malate and citrate. Furthermore, the 30-day pot experiment (Fig. 3c) showed that plant P uptake was similarly increased by both struvite and DAP (a positive control) when growing *F. esculentum*, which exudes organic acids from its roots in large quantities (Zheng et al. 1998). However, for *T. aestivum*, which does not exude large quantities of organic acids (Neumann and Römheld 1999), plant P uptake after struvite application remained at just 30 % of the level of that obtained after DAP application over the experimental period. It is also interesting that buckwheat mobilised significantly more P from this low P soil than spring wheat in the absence of applied fertiliser P (Fig. 3c).

### Plant P uptake and apparent fertiliser P recovery

**Figure 4:**
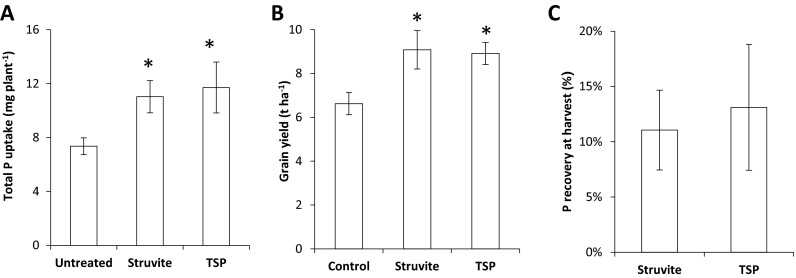
Struvite produces similar yield, P uptake and crop P recovery by *Triticum aestivum* to readily soluble P sources in a 90-day pot experiment. P was applied at 33.3 mg P pot^−1^ (35 kg P ha^−1^) in the form of struvite or triplesuperphosphate (TSP) alongside untreated controls. **a** The total P uptake resulting from each treatment, expressed in mg plant^−1^. **b** The grain yield, scaled up to t ha^−1^, of each treatment. **C** The P fertiliser recovery in the harvested plants. In A and B, asterisks mark values that are significantly different from the untreated negative controls using student’s t-test (*p* < 0.05). Error bars are standard errors of the mean

When *T. aestivum* was grown to harvest in the 90-day pot experiment, use of struvite produced very similar rates of total P uptake (Fig. 4a) and grain yield (Fig. 4b) per plant to those obtained with use of TSP. However the number of mature grain heads produced was significantly greater (*p* < 0.05) when TSP was applied (control =4.6 heads plant^−1^, struvite 4.8 heads plant^−1^, TSP 5.6 heads plant^−1^). Overall, crop P recovery was 11 % for struvite and 13 % for TSP (Fig. 4c). Any residue from the applied TSP granules could not be identified from the bulk soil and so has been assumed for these purposes to be completely dissolved. However, there were sizeable quantities of un-dissolved struvite after harvest ranging from 66 to 82 % of the initial mass. After subtracting the intact struvite granules which did not dissolve during the 90-day pot experiment, the apparent plant recovery of applied P from struvite (38 %) was 175 % greater than from TSP (13 %), even though the same P application rate was used (data not shown). This suggests that struvite has a considerable potential residual value for succeeding crops.

**Fig. 5:**
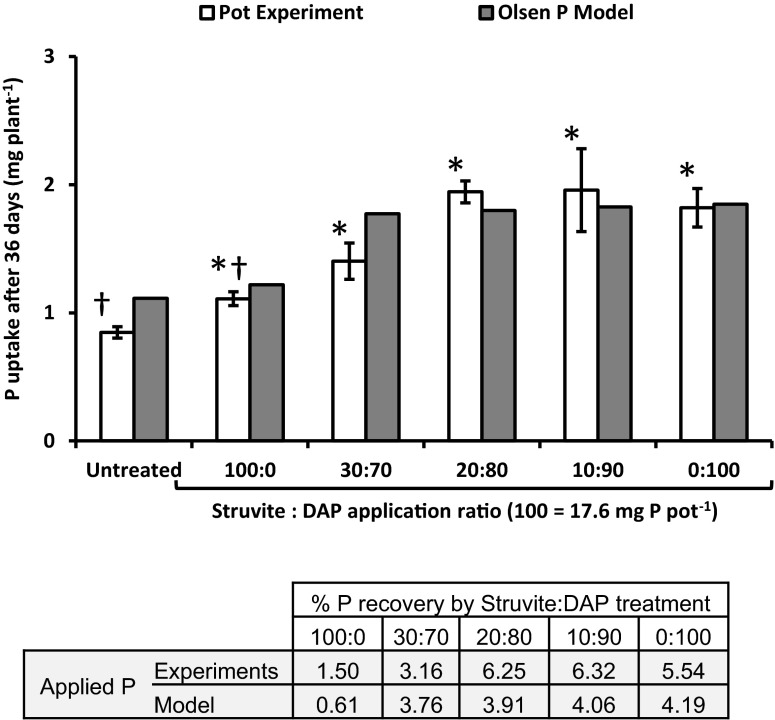
Mixtures of struvite and diammonium phosphate (DAP) improve P uptake relative to struvite alone in a 36-day pot experiment. Total P uptake by *Triticum aestivum* receiving 17.6 mg P pot^−1^ (35 kg P ha^−1^) as varying mixtures of struvite together with DAP alongside untreated controls (*white bars*). Asterisks mark pot trial values that are significantly different from the untreated negative controls, and daggers mark those that are significantly different from the 100 % DAP (0:100) positive control using student’s t-test (*p* < 0.05). Pot values of P uptake are compared with model simulations that used Olsen extractable P (grey bars) to calibrate the total plant available P in the soil. The recovery rate of the applied P for both the pot experiment and predicted by the model are given in the accompanying table. Error bars are standard errors of the mean

The 36-day pot experiment showed that there was a significant reduction (by 39 %) in early plant uptake of P by spring wheat using struvite compared to the readily soluble DAP fertiliser (Fig. 5). However, the use of mixtures of struvite with readily soluble P (where the struvite accounted for no more than 20 % of the total applied P) provided comparable levels of plant P uptake to the use of the readily soluble P fertiliser alone (Fig. 5). Hence 80 % of the P applied had to be in the form of DAP to maximise early growth and apparent crop P recovery when the fertiliser granules were placed in the soil. Maximum crop recovery of applied P was low at ca. 6 % (Fig. 5). As with the 90-day pot experiment, the plant recovery of struvite P alone (i.e. 100:0) was greatly increased (from 1.5 to 20 %) when the proportion of struvite granules which had actually dissolved was taken into account (data not shown). However, the greater dissolved P recovery of struvite did not improve the dissolved recovery rate of any of the struvite:DAP mixtures because of the dominance of DAP solubility in the mixture.

### Model predictions of P uptake

The outputs of the root P-uptake model for different combinations of struvite and DAP were generally consistent with the results of the 36-day pot trial regarding P uptake, confirming that placed struvite granules alone did not provide sufficient early plant P uptake compared with DAP alone, or mixtures including DAP (Fig. 5). However, whilst the pot experiment suggested at least 80 % DAP in the mixture was required for optimum plant P uptake, modelled data suggested this DAP contribution could be reduced to 70 % without loss in P uptake (Fig. 5). Modelled recovery rates of applied P were similar to the DAP only application for all struvite:DAP mixtures, and higher than for struvite alone. However, the model tended to under predict experimental values for P recovery, especially where DAP dominated the mixtures (Fig. 5).

**Fig. 6:**
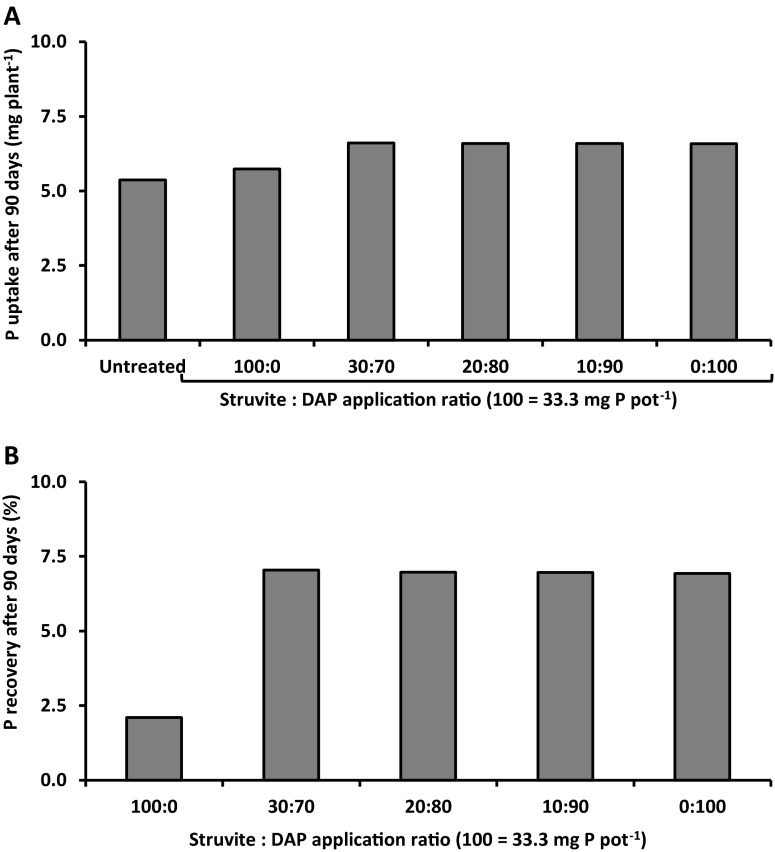
Model results showing the benefit of applying struvite together with diammonium phosphate (DAP) relative to struvite alone on P uptake and crop P recovery after 90 days by *Triticum aestivum* receiving 33.3 mg P pot^−1^ (35 kg P ha^−1^) and alongside untreated controls and a positive DAP only control. **a** The total P uptake resulting from each treatment, expressed in mg plant^−1^. **b** The recovery rate of P from the fertiliser granules at the end point of the experiment

When the model was run to grain harvest at 90 days, exactly the same trends were observed as after 36 days. Mixtures containing DAP resulted in a larger plant P uptake than for struvite alone (Fig. 6a). The higher P uptake for struvite alone compared with the untreated soil, and comparable P uptake to DAP alone, observed in the 90-day pot experiment (Fig. 4a) was therefore not predicted by the model. Instead only a very small increase was predicted (7 %) due to the slow dissolution rate for struvite; whereas there was an increase of 23 % in P uptake predicted by the model for DAP alone. This suggests that the slow dissolution of struvite by water was not the primary mechanism of P uptake. As in the 36-day model simulations, the 90-day model simulations predicted similarly low crop recoveries of the applied P for all mixtures containing DAP, and greater than for struvite alone (Fig. 6b).

## Discussion

### Struvite granules were a slow-release P fertiliser

The use of slow-release P fertilisers has the potential to contribute to more sustainable crop production systems operating under lower soil P fertility by reducing (a) soil P immobilisation processes and improving fertiliser P recovery, (b) the need for crops to allocate costly photosynthate to soil P acquisition strategies (extra root growth, exudates or mycorrhizas), and (c) the risk of fertiliser P loss in storm runoff directly after its application (Hart et al. 2004; Lambers et al. 2008; Withers et al. 2014b). Here we investigated whether commercial fertiliser grade struvite (i.e. spreadable granules) placed in the soil with the seed acted as a slow-release fertiliser, and how a greater understanding of struvite dissolution might inform a potential contribution to more sustainable crop production systems. When placed in the soil to maximise proximity to the growing root system, only a proportion of the struvite granules dissolved sufficiently to provide a source of P for early growth, yet P uptake after struvite application was similar to TSP application when spring wheat was grown to grain harvest in the 90-day pot experiment. At 36 days, only 9 % of the applied struvite had dissolved, but a much greater proportion had dissolved in the struvite-only treatment in the pot experiment taken to harvest at 90 days (26 %). X-ray tomography has previously shown that assessing the number of remaining intact granules is a suitable method for assessing the proportion of struvite P which has actually contributed to crop P uptake (Ahmed et al. 2015).

Although some caution is required in relating the results of pot experiments to field conditions, these results suggest that struvite granules do act as a slow release fertiliser compared to more soluble fertilisers, and that this slow rate of P release from struvite granules may be closer to the development of the crop root system’s capacity to take up P and may result in greater P-use efficiency (Sutton et al. 1983; Massey et al. 2009). The high proportion of intact granules remaining also suggests that struvite will have a residual value. To what extent this residual value differs from the residual value of TSP/DAP fertiliser after it has been immobilised by the soil remains to be evaluated. Hence, greater P recovery (and efficiency) from struvite can only be proven if the residual value of struvite proves to be greater than the residual value of TSP/DAP (Bonvin et al. 2015). This is likely to vary across different soil types depending on soil P binding strength and buffering capacity (Holford and Mattingly 1975; Sánchez 2010), and requires further research with labelled P. If substantiated by further research, the saving in P use over the crop rotation afforded by greater struvite efficiency will help to lower future pressure on finite reserves of PR.

### Struvite application alone does not allow sufficient early crop P uptake

Our results from the 36-day pot experiment showed that use of struvite alone produces lower rates of P uptake early in plant development than does the use of more readily soluble P fertilisers when these are placed in one location below the soil surface (Fig. 5). Early crop growth is important for establishing a well-developed root system, a resilient crop with optimum yield potential and for providing soil cover to prevent erosion (Pimentel et al. 1995; Grant et al. 2001; Flaval et al. 2014). The lower rates of crop P uptake from struvite during early growth are contrary to recent pot experiments where struvite was thoroughly mixed with the soil (Achat et al. 2014b; Bonvin et al. 2015), and there was no reduction in P uptake compared with soluble P fertiliser treatments. As soil pH was similar in all these studies, this suggests that either the struvite materials used were of a different physical composition to those in our study which affected solubility rates, or that placing struvite granules in one place in our experiments inhibited their solubility. Physical composition and surface area contact with soil are known to have a major influence on the P solubility of slow-release fertilisers (e.g. for raw PR products; Chien and Menon 1995). The synthetic struvite materials used in the studies of Achat et al. (2014b) and Bonvin et al. (2015) were either crushed crystals or fine powders, and very different to the hard fertiliser-grade 2.4 mm granules used in current commercial practice in the UK, and in our study. Placement is a well-recognised agronomic technique for enhancing root proliferation and P uptake in the rooting zone giving a yield advantage over broadcast application in low-P soils such as used here (Randall and Hoeft 1988; Flaval et al. 2014). However, the more limited surface area contact with the soil and associated microbial activity resulting from placement in one location may have reduced solubility rates sufficiently to restrict P uptake rates by spring wheat, especially as P acquisition by this crop is associated more with extension of its root system rather than release of organic acids (Ryan et al. 2014). This clearly requires further investigation.

Lower P uptake from struvite during early growth may also be a disadvantage for some yield components under field conditions. While in the 90-day pot experiment in controlled glasshouse conditions the P uptake and yield of grain obtained from plants fertilised with struvite alone was the same as that for plants fertilised only with soluble P fertiliser (Fig. 4a, b), the plants grown with struvite had a visibly reduced number of reproductive shoots and grain heads in later growth stages. The number of grain heads has long been recognised as both a very significant driver of final yield and to be determined at early growth stages (Brenchley 1929). This suggests that, in the pot experiment, the more sustained rate of P release from struvite facilitated carbohydrate translocation into the ripening grain leading to higher grain weights (Sutton et al. 1983), thereby compensating for fewer grain heads. Although the re-distribution of P already taken up by the plants meets a significant proportion of P demand for grain-filling, P-uptake from the soil at later growth stages may still be required to augment this (Boatwright and Haas 1961; Grant et al. 2001; Mohamed and Marshall 1979). To what extent this compensatory mechanism occurs when using struvite under field conditions of low soil P fertility remains unclear.

### Mixing struvite with a more soluble fertiliser P source has multiple advantages

Mixtures of struvite and readily soluble P fertilisers have the potential to couple the benefits of early P uptake levels of readily soluble P fertiliser with the more sustained slow P release from struvite that may benefit P uptake at later growth stages, and potentially increase overall fertiliser P recovery. These multiple benefits from struvite:DAP mixtures were evident in the 36-day pot experiment investigating early P uptake (Fig. 5) and the results of the model predictions for P uptake at 36-days and 90-days (Figs. 5 and 6). The model results indicated a potential gain in apparent P recovery rate after 90 days in the mixed treatments compared with use of the struvite alone (Fig. 6b), reflecting the limited dissolution of struvite compared to DAP. It is interesting to note that the model simulations, despite being calibrated using struvite dissolution rates in controlled conditions, consistently under-estimated the supply of P resulting from struvite treatment alone (Figs. 5 and 6a compared with Figs. 3c and 4a respectively). This under-estimation may be due to an increase in struvite dissolution rate when in close proximity to roots exuding organic acid anions, with the amount of struvite dissolved in the model simulations only reaching 53 % of the experimentally derived values at 36 days, and 55.4 % at 90 days. Although spring wheat is not a strong organic acid exuder, the model results indicate that interaction of struvite with soil and crop roots does increase its solubility. Similar conclusions were drawn by Ahmed et al. (2015) who used X-ray tomography to demonstrate increased dissolution of the same struvite granules in close proximity to a growing root.

The temporal variation in struvite release rates predicted by the model in the presence of varying amounts of DAP provides some indication of the optimum struvite:DAP fertiliser ratios for plant P uptake. For the struvite:DAP ratio (30:70), struvite release starts, stops within 1 day due to the high P concentrations from DAP dissolution and then starts again after about 16 days as the local P concentration decreases sufficiently due to soil P immobilisation and/or plant P uptake (Fig. 1a). This ratio is, in effect the approximate optimum, providing early P uptake to the root (from DAP) and maintaining P levels later on (as struvite). Higher DAP amounts would mean less, or none, of the struvite will dissolve, whilst lower amounts of DAP will allow struvite to continue to dissolve slowly over time, but, according to our measured data after 36 days (Fig. 5), would not provide enough P for early growth. The 36-day pot experiment suggested that a percentage of DAP in the mixture of at least 80 % was required to provide this early growth when the fertilisers were placed at 5 cm below the seed. Further development of the model is required to more closely match model predictions of P uptake dynamics to pot and field data.

### Struvite’s effectiveness as a P fertiliser may be enhanced for crop species that exude organic acids in large quantities

The results of the struvite solubility experiments showed a clear increase in both struvite’s P dissolution rate and the final solution equilibrium P concentration when treated with 1 mM of each of the organic acid anions tested (Fig. 3a). *Fagopyrum esculentum* also proved to be significantly more effective at taking up P after struvite fertilisation than *T. aestivum*, a result that could be attributed to its higher rate of organic acid exudation (Zhang et al. 2001; Zheng et al. 1998). The *F. esculentum* root system is known to exude large quantities of oxalic acid even when unstressed (Zheng et al. 1998), and oxalic acid had the biggest impact on struvite solubility of the organic acids tested in our experiment (Fig. 3b). Surprisingly, there was no clear relationship between struvite P dissolution rate and the stability constants of the different organic anion-Mg^2+^ complexes. This suggests that the enhanced dissolution is unrelated to lowering the Mg^2+^ concentration in the external solution, but probably relates to a direct surface interaction. Our results show the potential benefit of struvite use on other commercially valuable crops whose root systems also exude organic acids in large quantities: this includes *Brassica napus*, *Cicer arietinum* and *Lupinus albus* all of which exude large quantities of malate and citrate into the rhizosphere reaching concentrations in the low mM range (i.e. within the range used in the struvite dissolution experiments undertaken here; Ligaba et al. 2004; Dessureault-Rompre et al. 2006) (Fig. 3b). It is also likely that there is a positive interaction between H^+^-ATPase-mediated H^+^ release and organic acid anion release in terms of enhanced struvite dissolution and this requires further investigation in a rhizosphere context. To what extent lower organic anion concentrations in soils (5–50 μM) produced by other common crops (e.g. *Triticum aestivum*, *Zea mays*) would achieve the same dissolution effect on struvite requires further work. This is especially pertinent given that these concentrations typically have minimal effect on mineral dissolution rates (Jones 1998; Drever and Stillings 1997). However, in high exuding crops, this interaction potentially creates a specific advantage of struvite over conventional fertilisers for sustainable nutrition of many crops, especially in low P soils (Withers et al. 2014b). As there was no significant increase in struvite solubility when an infinite (resin) P sink was included in the laboratory assay, root-derived organic acids may be destabilising the surface of struvite granules directly rather than a solution complexation-based reaction. A slow-release fertiliser that actively responds to the presence of a crop root system (e.g. Ahmed et al. 2015) has the potential to be a far more spatially precise, efficient method of fertilising plants with P than application of conventional, highly soluble P fertilisers and may also eliminate the need for mixing struvite with readily soluble P fertiliser to fulfil the crop’s early P uptake demands.

### Soil pH, Mg^2+^ and NH_4_^+^ concentrations are unlikely to be detrimental to struvite P-fertilisation

The present study confirmed the expected result (Bhuiyan et al. 2007; Massey et al. 2009) that the initial solubility of struvite was increased by a reduction in pH, but that the final equilibrium P concentration was unaffected by pH (Fig. 2). When applied to soil in combination with readily soluble P fertilisers, the initial P dissolution rate of struvite is rendered unimportant. The soil solution P concentration will rapidly far exceed the point at which struvite dissolution is arrested (Fig. 1a) until either soil P-fixation, leaching or plant uptake removes sufficient of the dissolved P from solution. An important factor in the use of mixed fertilisers is the equilibrium P concentration that struvite can maintain later in the growing season, once the effects of the readily soluble fertiliser P have diminished. This is unlikely to be significantly impacted by soil solution pH for this kind of fertiliser mix. Our pot experiments used only soils with a pH of 6.0, but Massey et al. (2009) have previously shown that struvite fertiliser is effective in moderately alkaline soils (pH 7.6), which adds further evidence that within the range found in agricultural soils pH does not have a significant impact upon struvite effectiveness over a growing season. Achat et al*.* (2014a) also found that soil pH did not influence the effectiveness of struvite in a series of incubation experiments. Experimentation on the effects of the presence of counter-ions on struvite P dissolution found that solution NH_4_^+^ concentration had a negative effect on the equilibrium P concentration (Supplementary Figure 1). However, this effect was only small over the wide range of concentrations tested, so it is unlikely to be an important consideration when planning fertiliser strategies.

## Conclusions

This study has shown that placement of commercially available struvite granules significantly altered the pattern of plant P uptake during the growing season relative to placement of highly-soluble inorganic P fertilisers. The slower rate of P release from struvite granules reduced plant uptake of P during early growth but without detriment to final yield. Positive impacts on fertiliser P recovery by the crop were obtained if calculated relative to the small proportion (up to 26 %) of struvite that actually dissolved during the growing season. Mixtures of struvite and readily soluble P show promise as a more sustainable fertiliser strategy than sole use of either fertiliser by maximising early crop nutrition, whilst also supplying P at later stages of plant development when P demand is at its peak, and providing a potential source of residual P available for subsequent crops. Our experimental evidence indicates that organic acids have a major impact on the rate of dissolution of P from struvite and plant species with root systems that exude large quantities of organic acids will be much more effective at taking up P from struvite granules. Therefore struvite has an especially high potential for spatial and temporal targeting of P for root uptake for such crops. Further field experimentation is now required to assess the effectiveness of these proposed P fertiliser strategies under field conditions, for a wider range of soil types and cropping systems.

## Electronic supplementary material

Supplementary Figure 1 Images of intact 36-day-old *T. aestivum* root systems grown in loamy sand soil (Olsen-P = 13 mg kg^−1^) without applied P (low P) or with diammonium phosphate (DAP) placed at 5 cm below the seed (high P) in pots. Maximum root branching rates were calculated from these images for use in the root P uptake model. (PPTX 856 kb)

Supplementary Figure 2 The effects of environmental counter-ion concentration on struvite P dissolution. Struvite granules of 2.4 mm diameter were submerged in 1 ml solutions containing 0–1000 μM concentrations of either NH_4_^+^ (A, D), Mg^2+^ (B, E), or PO_4_^3−^ (C, F). There were three replicates per treatment. The concentration of solution P was measured over time, and the curve f(x) = a(1-b^x^) was then fitted to each replicate individually. This was used to calculate their initial P dissolution rate (A-C), and final equilibrium P concentration (D-F). The Pearson product-moment correlation coefficient for both datasets was calculated: showing strong negative correlations of: initial [Pi] with P dissolution rate (C), initial [NH_4_^+^] with equilibrium [Pi] (D), and initial [Pi] with equilibrium [Pi] (F). The values on the y-axis in panel C represent only struvite-derived P concentration in solution, not total solution P. (PPTX 74 kb)
