# Supplementary figures and images for: Struvite: a slow-release fertiliser for sustainable phosphorus management?
Source: Plant Soil. 2015 Dec 11;401:109–23. doi: 10.1007/s11104-015-2747-3 (PMC4923718; doi:10.1007/s11104-015-2747-3)

## Slide 1
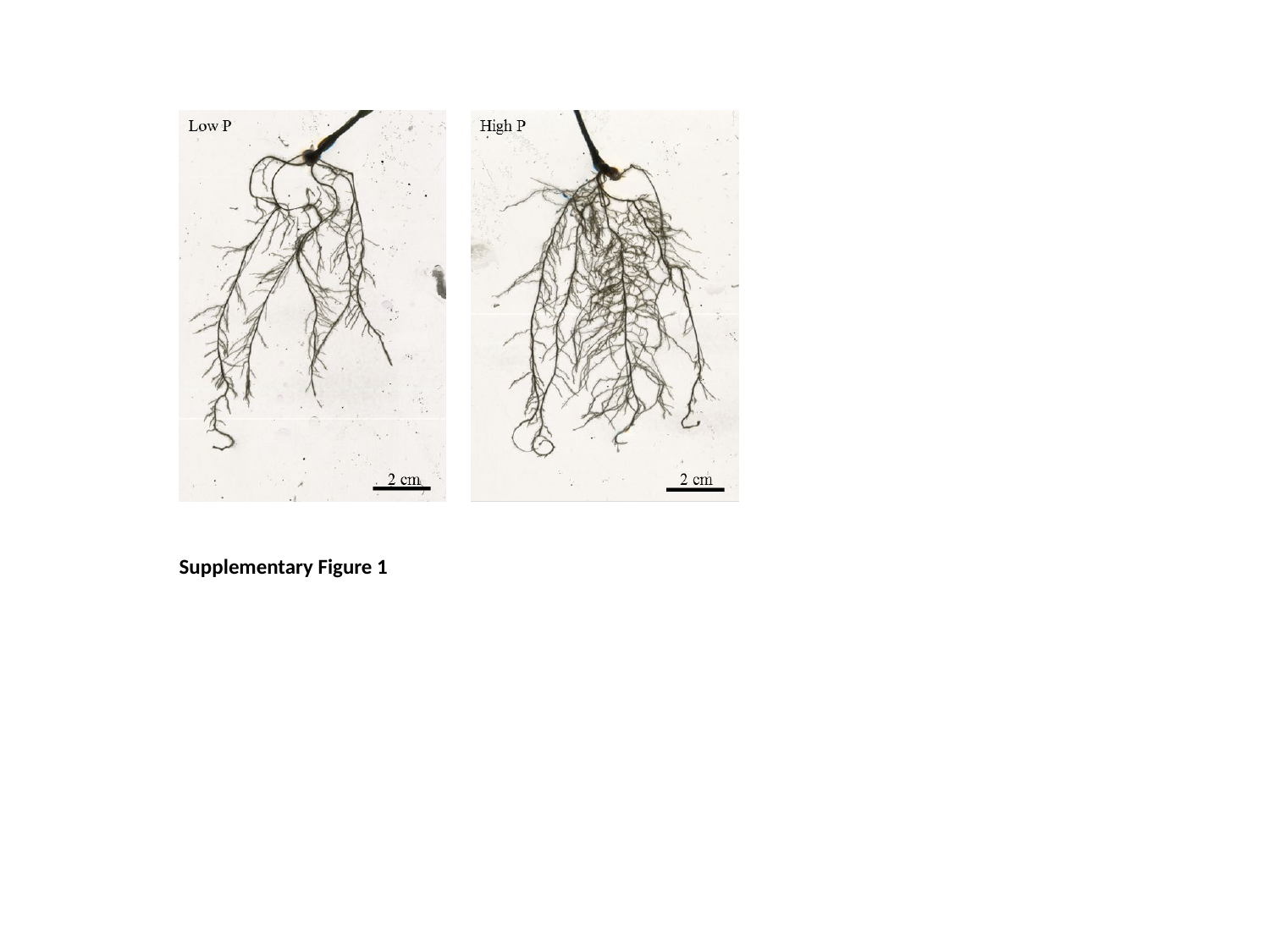

Supplementary Figure 1

Supplement: Supplementary file 1 — Images of intact 36-day-old T. aestivum root systems grown in loamy sand soil (Olsen-P = 13 mg kg−1) without applied P (low P) or with diammonium phosphate (DAP) placed at 5 cm below the seed (high P) in pots. Maximum root branching rates were calculated from these images for use in the root P uptake model. (PPTX 856 kb) [file 11104_2015_2747_MOESM1_ESM.pptx]
