# Supplementary material for: Struvite: a slow-release fertiliser for sustainable phosphorus management?
Source: Plant Soil. 2015 Dec 11;401:109–23. doi: 10.1007/s11104-015-2747-3 (PMC4923718; doi:10.1007/s11104-015-2747-3)
Supplement: Supplementary file 2 — The effects of environmental counter-ion concentration on struvite P dissolution. Struvite granules of 2.4 mm diameter were submerged in 1 ml solutions containing 0–1000 μM concentrations of either NH4 + (A, D), Mg2+ (B, E), or PO4 3− (C, F). There were three replicates per treatment. The concentration of solution P was measured over time, and the curve f(x) = a(1-bx) was then fitted to each replicate individually. This was used to calculate their initial P dissolution rate (A-C), and final equilibrium P concentration (D-F). The Pearson product-moment correlation coefficient for both datasets was calculated: showing strong negative correlations of: initial [Pi] with P dissolution rate (C), initial [NH4 +] with equilibrium [Pi] (D), and initial [Pi] with equilibrium [Pi] (F). The values on the y-axis in panel C represent only struvite-derived P concentration in solution, not total solution P. (PPTX 74 kb) [file 11104_2015_2747_MOESM2_ESM.pptx]

## Slide 1
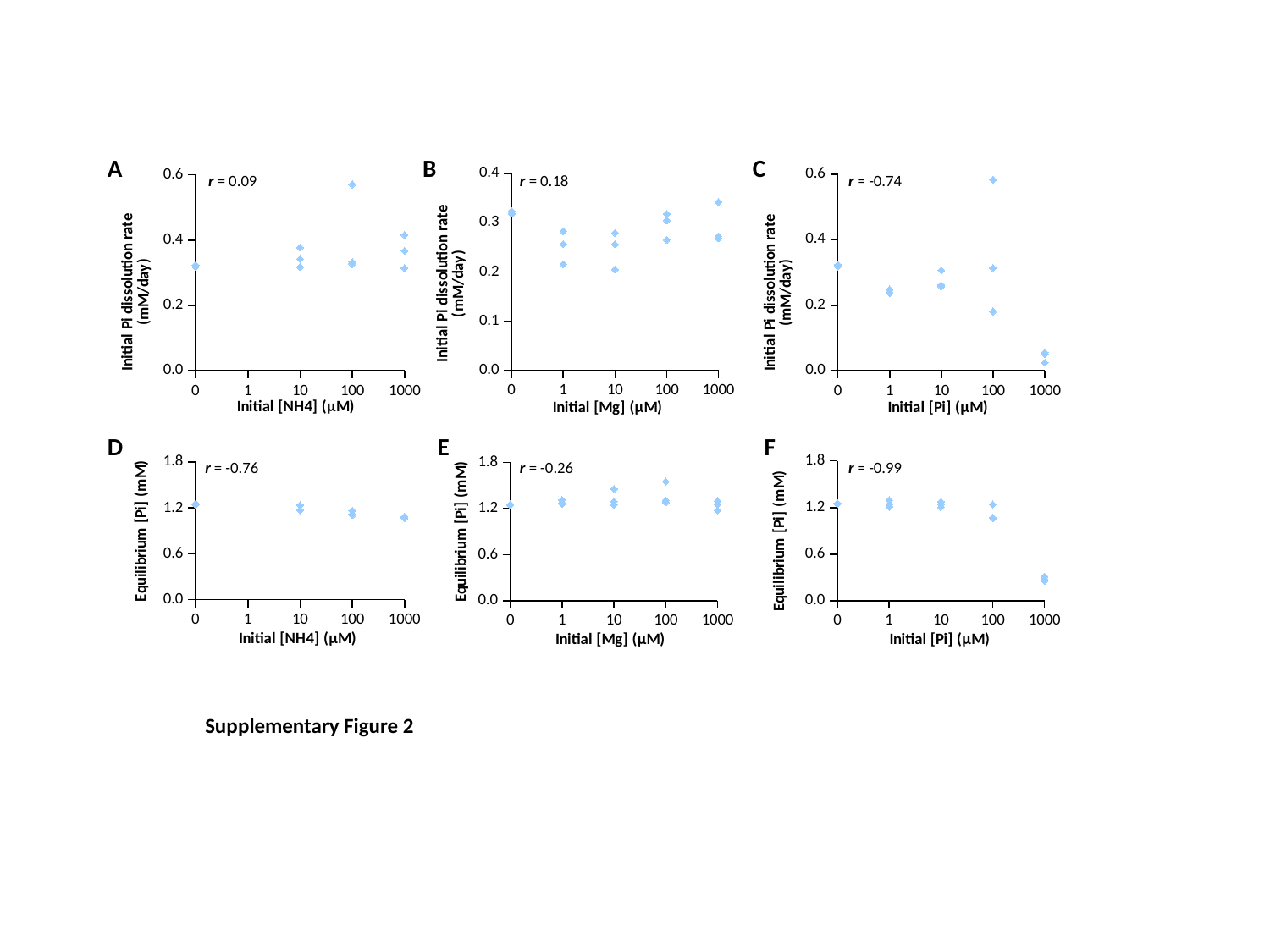

### Chart
| Category | |
|---|---|
### Chart
| Category | |
|---|---|A
B
C
### Chart
| Category | |
|---|---|r = 0.09
r = 0.18
r = -0.74
E
D
F
### Chart
| Category | |
|---|---|
### Chart
| Category | |
|---|---|
### Chart
| Category | |
|---|---|r = -0.76
r = -0.26
r = -0.99
Supplementary Figure 2
